# MEDIATOR 25: A missing link in tomato ripening

**DOI:** 10.1093/plcell/koad015

**Published:** 2023-01-21

**Authors:** Sara Lopez-Gomollon

**Affiliations:** Assistant Features Editor, The Plant Cell, American Society of Plant Biologists, USA; Department of Plant Sciences, University of Cambridge, Cambridge CB23EA, UK

Some plants have evolved an exquisite strategy to ensure the survival of their species. Seeds are wrapped in attractive and nutritious fruits, so animals get nourishment while plants get their seeds dispersed. Ripening is the biochemical and physical changes that transform an unappealing fruit protecting the developing seeds into a desirable snack full of seeds ready to germinate. In fruits like tomato or banana, a burst of ethylene and a plethora of transcriptional factors trigger a cascade of changes in gene expression to produce a soft, aromatic, and colorful ripe fruit (Fenn and Giovannoni, 2021).

**Figure:**
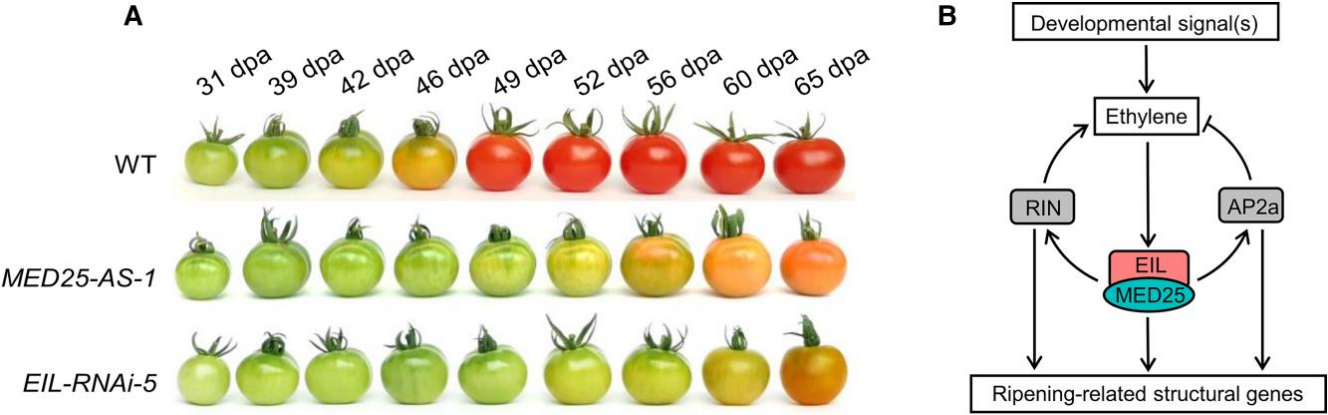
Tomato MED25 regulates fruit ripening by interacting with EIN3-like transcription factors. A, Knockdowns of *MED25* and *EIL1-4* in tomato (*Solanum lycopersicum*) impairs fruit ripening (dpa—days post anthesis). B, Working model showing the role of MED25-EIL in regulating fruit ripening. Adapted from Deng, Yang, Li, Chang et al. (2022), Figures 1 and 7.

But how do transcriptional factors connect to the transcriptional machinery to change THE expression of ripening genes? This is the question that **Lei Deng, Tianxia Yang, Qian Li, Zeqian Chang, and colleagues** (Deng et al., 2022) set out to answer, using tomato as a model system. The authors postulated the missing link to be Mediator (MED), a conserved oligomeric protein complex known for enabling the interaction between transcriptional factors and RNA polymerase (Pol) II (Allen and Taatjes, 2015). However, there are 33 subunits in the tomato genome, eight of which are encoded by homologous genes. The authors silenced the expression of several MED proteins in stable transgenic lines, finding a delay in fruit ripening only when MED25 was targeted (see Figure). Key changes associated with tomato ripening include red color and fruit softening; and indeed the enzymes involved in those processes (lycopene production, chlorophyll degradation, cell wall remodeling) were affected in the MED25 silenced line, supporting its role in regulating fruit ripening.

The authors found that the MED25 silenced line is impaired in both the autocatalytic synthesis of ethylene and the expression of ripening-related transcription factors, suggesting that MED25 recruits ethylene-responsive and/or ripening transcriptional factors to Pol II. Using yeast-two-hybrid assays, the authors identified four proteins from the ETHYLENE-INSENSITIVE 3 (EIN3)/EIN3-LIKE (EIL) family able to interact with MED25 (Merchante et al., 2013). Transgenic knockout lines for *EIL1-4* confirmed that these proteins are required for ripening-induced ethylene biosynthesis (see Figure).

If MED25 facilitates the transcriptional regulation of EIL1-4, we would expect that knocking out either one would affect the expression of a similar set of genes. RNAseq data showed that 60.7% of the ripening-related genes are co-regulated by MED25 and EIL1-4. The binding sequences of the MED25-EIL1 complex were then identified using “CUT&Tag” (Kaya-Okur et al., 2019). In this technique, the DNA binding region of a protein (EIL1 in this case) is recognized by an antibody recruiting a modified transposase that will cut close to the binding site while adding primers for next-generation sequencing. Most binding sites identified corresponded to gene promoters, but only 19% (1,110 genes) were identified as EIL-regulated ripening genes by RNAseq, suggesting that EIL1 indirectly regulates the remaining genes. The authors then compared these 1,110 genes with the MED-regulated genes previously identified by RNAseq and found a majority (87%) regulated by both EIL and MED25, strongly indicating that EIL and MED25 form a transcriptional complex to regulate ripening-related genes. This group included both up-regulated and down-regulated genes, implying that the complex can act as an activator or repressor of gene expression. The functions of the regulated genes were both structural (fruit ripening-related) and regulatory (related to modulation gene expression). The regulatory genes included both activators and repressors of ethylene biosynthesis, suggesting that the MED25-EIL complex maintains ethylene homeostasis during ripening (see Figure).

Fruit ripening depends on the tight temporal control of ripening-related genes. Several transcriptional factors were identified as key for this process, but how the ripening-specific transcriptional regulators communicate with the Pol II general transcriptional machinery was not known. In this work, the authors demonstrated that MED25 physically interacts with EIL transcription factors, transmitting the ethylene signal to the Pol II transcriptional machinery to regulate the expression of ripening-related genes. Future research could further identify the mechanisms and cofactors enabling the MED25-EIL complex to act as an activator for some genes but as a repressor for others.
